# Editorial: Mindfulness in interpersonal, group, organization, media, and cross-culture communication

**DOI:** 10.3389/fpsyg.2024.1429978

**Published:** 2024-05-27

**Authors:** Hao Chen, Chao Liu, Wen-Ko Chiou

**Affiliations:** ^1^School of Film Television and Communication, Xiamen University of Technology, Xiamen, China; ^2^School of Journalism and Communication, Hua Qiao University, Xiamen, China; ^3^Department of Industrial Engineering and Management, Ming Chi University of Technology, New Taipei, Taiwan; ^4^Department of Psychiatry, Chang Gung Memorial Hospital, Taoyuan, Taiwan; ^5^Department of Industrial Design, Chang Gung University, Taoyuan, Taiwan

**Keywords:** mindfulness, interpersonal communication, group communication, media communication, cross-culture communication

## Introduction

In the dynamic field of communication studies, the infusion of mindfulness has emerged as a transformative force, influencing interactions at all levels: interpersonal, group, organizational, media, and cross-cultural. This Research Topic, “*Mindfulness in Interpersonal, Group, Organization, Media, and Cross-culture Communication*,” seeks to explore and illuminate these intersections, presenting a rich tapestry of research that highlights how mindfulness can profoundly enhance understanding and effectiveness in diverse communication settings.

Mindfulness, often understood as the practice of being present and fully engaged with the moment without judgment, has far-reaching implications for communication. At the interpersonal level, it fosters deeper empathy and more authentic connections, allowing individuals to respond rather than react to others. Within groups and organizations, mindfulness aids in reducing conflict and enhancing collaboration by helping members attend more fully to each other's ideas and emotions. In media contexts, mindfulness can influence both the creation and reception of content, encouraging a more thoughtful and discerning approach to information consumption and production. Finally, in cross-cultural settings, mindfulness provides valuable tools for navigating the complexities of intercultural misunderstandings and embracing differences with respect and curiosity.

This editorial aims to synthesize the insights from four pivotal articles featured in this Research Topic. Each article offers a unique lens on the intersection of mindfulness and communication, covering therapeutic settings, educational resilience, cultural dynamics, and technological innovations. By examining these contributions, this editorial will draw out overarching themes, discuss practical implications, and suggest directions for future research, aiming to bridge the gap between theoretical exploration and practical application in the field of mindful communication.

## Article summaries and analyses

“*Positive communication workshops: are they useful for treatment programmes for anorexia nervosa?”*

This article delves deep into the application of mindfulness in therapeutic communication, particularly within the sensitive and complex context of treating anorexia nervosa. The study investigates the effectiveness of positive communication workshops in enhancing treatment outcomes for individuals battling this condition. Through a combination of qualitative and quantitative methods, the research reveals that mindfulness-oriented communication strategies can significantly improve patient engagement and recovery rates. These workshops, incorporating principles of mindful listening and speaking, enable therapists and patients to establish a more empathetic and supportive therapeutic relationship. The implications for healthcare professionals are profound, as this suggests that integrating mindfulness into communication can lead to more effective, compassionate care, potentially transforming treatment paradigms in mental health settings (Tchanturia et al.).

“*Progressive muscle relaxation in pandemic times: bolstering medical student resilience through IPRMP and Gagne's model*”

In this article, the focus shifts to the educational sector, where the pressures of the pandemic have heavily impacted medical students' mental health and communication skills. The research explores how progressive muscle relaxation, guided by mindfulness principles and Gagne's instructional model, can bolster resilience among students. This intervention was tested in a longitudinal study with medical students experiencing heightened stress due to the pandemic's uncertainties and demands. The outcomes highlight not only improved stress management but also enhanced interpersonal communication, which is critical for future medical practitioners. By facilitating a clearer mind and reduced anxiety, these mindfulness practices help students engage more fully in their learning and interactions, suggesting a broader application for mindfulness in educational resilience and professional development (Nair et al.).

“*Collectivism, face concern and Chinese-style lurking among university students: the moderating role of trait mindfulness*”

This intriguing article examines the complex dance between cultural communication norms and mindfulness. Focusing on Chinese university students, the study explores how trait mindfulness moderates behaviors like “face concern” and “lurking” in digital communication environments. Through a detailed analysis, the research underscores the potential of mindfulness to alter traditional communication patterns, fostering more open and authentic interactions even within rigid cultural frameworks. The findings reveal that students with higher levels of trait mindfulness are less likely to engage in lurking behavior and are more adept at navigating the cultural imperative of “face,” leading to more direct and honest communication in online settings. This has significant implications for understanding how mindfulness can serve as a bridge in cross-cultural communication, facilitating greater understanding and cooperation among diverse populations (Hu et al.).

“*How flow and mindfulness interact with each other in mindfulness-based augmented reality mandala coloring activities*”

The final article featured in this Research Topic bridges the gap between technology and mindfulness. It investigates how augmented reality (AR) mandala coloring activities can facilitate a state of flow and mindfulness, enhancing the overall communication experience. This study provides a fascinating glimpse into how digital tools can be used to promote mindfulness, with significant implications for designing media content that supports mindful engagement and interaction. The research demonstrated that participants engaging in AR mandala coloring experienced increased levels of flow and mindfulness, leading to improved mood, and reduced anxiety. This suggests that integrating mindfulness principles into digital and media design can create more engaging, restorative experiences that support mental health and wellbeing (Chen et al.).

## Synthesis of emerging themes

From the comprehensive examination of the articles, several key themes emerge that highlight the transformative power of mindfulness in communication:

Enhancement of Empathy and Authenticity: Across all contexts, from therapeutic sessions to educational environments, mindfulness fosters deeper empathy and authenticity in communication. This is seen in how therapists and patients, as well as teachers and students, connect more meaningfully when mindfulness is part of their interaction.

Resilience in High-Stress Environments: The studies demonstrate that mindfulness tools, such as progressive muscle relaxation, help individuals in high-stress professions like healthcare and education navigate stress and maintain clear, effective communication. This resilience is crucial for sustaining performance and wellbeing in challenging times.

Cultural Adaptability: Mindfulness can help modify traditional communication behaviors, making them more adaptable to diverse cultural settings. The study on Chinese university students shows that mindfulness can soften rigid cultural communication norms, promoting more open and inclusive interactions.

Technological Integration: The use of digital tools like AR in promoting mindfulness illustrates the potential for technology to enhance, rather than detract from, meaningful communication. This opens up exciting possibilities for using technology to support mindfulness and improve overall communication quality.

## Implications for practice

The practical applications of these insights are vast. In healthcare, mindfulness can be integrated into patient-provider interactions to improve treatment outcomes. In education, mindfulness exercises like progressive muscle relaxation can be used to enhance students' resilience and communication skills. Culturally, mindfulness can help bridge gaps in digital communication, particularly among youth navigating globalized online spaces. Technologically, the development of AR and other digital tools should consider mindfulness principles to foster more engaged and reflective user experiences.

## Future research directions

While this Research Topic has broadened our understanding of mindfulness in communication, there remain numerous avenues for further research. Future studies could explore mindfulness in other high-stress professions, examine its role in different cultural contexts, or delve deeper into the interaction between mindfulness and emerging communication technologies.

## Conclusion

The contributions of this Research Topic underscore the vital role of mindfulness in transforming communication across various spheres. As we continue to navigate complex interpersonal, organizational, and cultural landscapes, mindfulness offers a key to more effective, empathetic, and authentic communication.

## Author contributions

HC: Writing – original draft. CL: Conceptualization, Methodology, Writing – review & editing. W-KC: Supervision, Writing – review & editing.

